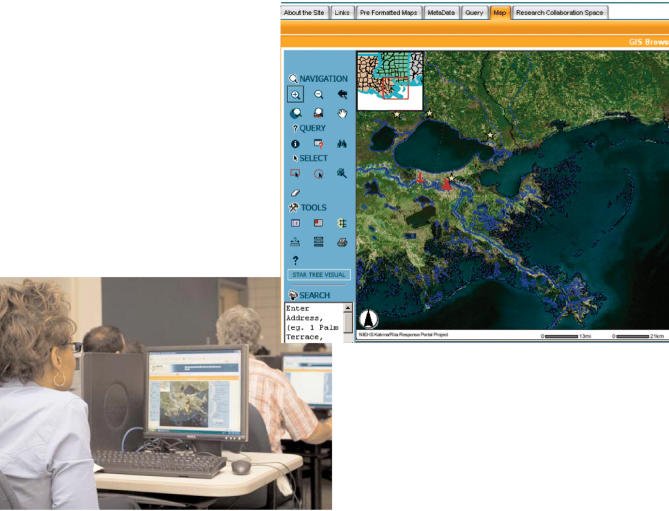# Update on the NIEHS Hurricane Response Portal

**Published:** 2006-09

In the aftermath of devastation of the Gulf Coast, teams of NIEHS staff responded by mobilizing resources. Within the Division of Extramural Research and Training, a group of Superfund Basic Research Program grantees pooled resources to create an interactive, web-based geographic information system (GIS) portal that provides the ability to share information, software, and computer processes across organizational boundaries. The NIEHS Environmental Health Science Data Resource Portal allows users to access demographic, public health, infrastructure, and environmental data, all of which are fully georeferenced in a user-friendly and highly customizable research environment (see http://www.apps.niehs.nih.gov/Katrina/). The spatial data sets incorporated into the portal contain basic infrastructure data such as those on roads and electric power plants, potential contaminant sources including Superfund and Toxic Release Inventory sites, hurricane flooding data, Census data, physiographic data, and remote sensing imagery both pre- and post-Katrina.

This portal is a collaborative effort, drawing expertise from the University of California at San Diego, Duke University, Columbia University, University of Kentucky, Research Triangle Institute, and San Diego State University, with input from local government, housing, and community groups in the Gulf Coast region. The goal is to provide resources needed to monitor and evaluate the human health impacts of the hurricane events; assess/reduce human exposures to contaminants; and develop science-based remediation, rebuilding, and repopulation strategies.

The portal environment provides the research community with a flexible framework in which to collaborate and conduct analysis and visualization using a variety of data sets and web-based tools. Parts of the portal are open to the public. Secured areas can be used by researchers to upload and integrate their data into the existing data sets available within the portal. If interested in learning how the portal can be customized to meet your needs, contact hurricanegis@niehs.nih.gov.

The NIEHS Environmental Health Science Data Resource Portal adds to the previously reported activities of the NIEHS Worker Education and Training Program grantees (Extramural Update. Environ Health Perspect 114:A115). This group developed a hazard awareness orientation PowerPoint presentation and booklet to provide field-accessible safety awareness information in a worker-friendly format.

Both the portal and the hazardous awareness orientation materials were designed to provide access to critical and timely information in the event of similar disasters in the future.

## Contacts

**Claudia Thompson, Ph.D. |** thomps14@niehs.nih.gov

**Beth Anderson |** tainer@niehs.nih.gov

## Figures and Tables

**Figure:**